# Performance of Real-Time Elastography for the Staging of Hepatic Fibrosis: A Meta-Analysis

**DOI:** 10.1371/journal.pone.0115702

**Published:** 2014-12-26

**Authors:** Huisuo Hong, Jia Li, Yin Jin, Qiao Li, Weimin Li, Jiansheng Wu, Zhiming Huang

**Affiliations:** 1 Department of Gastroenterology, First Affiliated Hospital of Wenzhou Medical University, Wenzhou, Zhejiang Province, China; 2 Ultrasound Imaging Center, First Affiliated Hospital of Wenzhou Medical University, Wenzhou, Zhejiang Province, China; Yonsei University College of Medicine, Republic Of Korea

## Abstract

**Background:**

With the rapid development of real-time elastography (RTE), a variety of measuring methods have been developed for the assessment of hepatic fibrosis. We evaluated the overall performance of four methods based on RTE by performing meta-analysis of published literature.

**Methods:**

Online journal databases and a manual search from April 2000 to April 2014 were used. Studies from different databases that meet inclusion criteria were enrolled. The statistical analysis was performed using a random-effects model and fixed-effects model for the overall effectiveness of RTE. The area under the receiver operating characteristic curve (AUROC) was calculated for various means. Fagan plot analysis was used to estimate the clinical utility of RTE, and the heterogeneity of the studies was explored with meta-regression analysis.

**Results:**

Thirteen studies from published articles were enrolled and analyzed. The combined AUROC of the liver fibrosis index (LFI) for the evaluation of significant fibrosis (F≥2), advanced fibrosis (F≥3), and cirrhosis (F = 4) were 0.79, 0.94, and 0.85, respectively. The AUROC of the elasticity index (EI) ranged from 0.75 to 0.92 for F≥2 and 0.66 to 0.85 for F = 4. The overall AUROC of the elastic ratio of the liver for the intrahepatic venous vessels were 0.94, 0.93, and 0.96, respectively. The AUROC of the elastic ratio of the liver for the intercostal muscle in diagnosing advanced fibrosis and cirrhosis were 0.96 and 0.92, respectively. There was significant heterogeneity in the diagnostic odds ratio (DOR) for F≥2 of LFI mainly due to etiology (*p*<0.01).

**Conclusion:**

The elastic ratio of the liver for the intrahepatic vein has excellent precision in differentiating each stage of hepatic fibrosis and is recommend to be applied to the clinic.

## Introduction

Hepatic fibrosis, which occurs in patients suffering from chronic liver diseases (CLDs), is a pathological process [1] that is characterized by an accumulation of extracellular matrix (ECM). Without appropriate and timely intervention, progressive hepatic fibrosis will gradually lead to cirrhosis, hepatocellular carcinoma, and finally liver failure [2], [3]. Therefore, the precise diagnosis and assessment of liver fibrosis is crucial for the prevention, prognosis, surveillance, and optimization of treatment strategies in CLD patients [4], [5].

Currently, liver biopsy (LB) is recommended as a reference standard to assess the degree of liver fibrosis [6]–[8]; however, LB is an invasive procedure possibly leading to patients' discomfort. Moreover, LB is susceptible to sampling errors and significant intra- and inter- observer variability [9], [10], and may cause serious complications, such as fatal bleeding, biology related mortality and other limits [8]. Therefore, non-invasive methods have been proposed to assess the severity of hepatic fibrosis as alternatives to biopsy [11], [12], including serum biomarkers, scoring systems and image examination. Among these non-invasive methods, ultrasound elastography which measures the stiffness of the liver related to hepatic fibrosis [13], has been explored. Transient elastography (TE), Real-time Elastography (RTE), Acoustic Radiation Force Impulse Imaging (ARFI) and Shear Wave Elastography (SWE) have all been evaluated for their role in assessing the degree of hepatic fibrosis [14]–[17].

The calculation processes and results are different among various methods, however, TE, ARFI and SWE measure the speed of the shear wave related to the liver elasticity. The elastic shear wave is generated by an acoustic pulse or a vibration and propagates through the tissue examined. Different from the three methods above, RTE diagnoses hepatic fibrosis based on the strain within the tissue created by external compression. In recent years, RTE has received increasing attention and has been studied in a multitude of liver diseases that exhibit different stages of liver fibrosis. As a new ultrasonic technology for the evaluation of hepatic fibrosis, researchers around the world are exploring various methods based on RTE to replace LB in the clinic. However, varying results have been reported in published studies using different quantitative methods based on RTE, which limits the clinical use of RTE [15], [18]–[20]. A systematic approach is required for integrating the RTE data from independent studies to evaluate the role of each method in assessing hepatic fibrosis, thus we performed a meta-analysis to provide a combined systematic review of the accuracy of different quantitative RTE methods in patients with CLDs and evaluated the clinical utility of these methods.

## Materials and Methods

### Real-Time Elastography

RTE is a novel technology for obtaining images using ultrasound. The principle of the RTE technique is based on slight external tissue compression on the structures examined, which produces strain (displacement) within the tissue and allows for subsequent calculation of the strain profile along the axis of compression [21]. The equipment measures mechanical deformation (strain) of tissues, generating color-coded maps of the strain distribution (elastograms) overlapping B-mode image, which reflect tissue elasticity. The color ranges from red to blue to show the relative stiffness of the area inside the region of interest (ROI). The harder areas are displayed in blue and the softer areas in red. However, the strain image is a qualitative expression and not a quantitative evaluation for liver stiffness. With the development of RTE, different quantitative methods that measure the stiffness of the liver were validated in previous studies, as described below:

The first quantitative method called liver fibrosis index (LFI). In this method, the internal compression and relaxation of the ROI induced by the external compression forms two consecutive frames and the ultrasound system obtains the strain of the tissue to construct the RTE image. Then, the software extracts nine parameters to characterize the RTE image. These parameters were often applied in many fields, such as satellite imaging, geothermal imaging and machine visions. LFI is estimated using the nine parameters, which characterize the elastogram, as independent variables and the hepatic fibrosis stage as a dependent variable within a multiple regression equation [22].

The second quantitative method called elasticity index (EI). Compared to LFI, another equation was studied by Colombo et al. and Juan Wang et al. [19], [23]. Different from LFI, they generated 11 parameters obtained via RTE. Subsequently, elasticity index (EI) was calculated using the 11 parameters in a multiple regression equation.

Without using different formulas to measure hepatic fibrosis, some studies simultaneously chose the intrahepatic venous small vessels and the hepatic parenchyma as two ROIs and calculated the strain of each distribution. The elastic ratio (ER) was then defined as the value of the intrahepatic venous small vessels divided by the value of the hepatic parenchyma [15]. The elastic ratio is defined as ER1 in this article for convenience.

In other studies, Xie et al. and Paparo et al. defined the elastic ratio as the value of perihepatic soft-tissues (diaphragm and intercostal muscles) divided by the value of the hepatic parenchyma [20], [24]. They recommend the use of the soft-tissues as internal control since the soft-tissues appeared quite homogeneously soft (when compared to the liver parenchyma) in all patients. The elastic ratio is defined as ER2 in this article for convenience.

There are several other RTE methods we did not discuss in this article because few studies utilized these methods. [25], [26]

### Literature Search

A computerized search was performed in PubMed (MEDLINE), the Cochrane library, EMBASE, and Google Scholar to identify relevant articles published from April 2000 to April 2014. The following terms were used: real time elastography, fibrosis, liver, hepatic, ultrasound, elastography. And we did not have a protocol existed for our meta-analysis.

### Inclusion and Exclusion Criteria

The full text of every article was scrutinized to determine whether they were original studies. Then, the full article was further assessed according to the following inclusion/exclusion criteria. Inclusion/exclusion criteria required the following features:

- The study evaluated the performance of RTE in assessing liver fibrosis stages due to various liver diseases, with quantitative measurement.
- Liver biopsy or TE was recommended as the reference standard. Fibrosis staging was based on the METAVAIR scoring system, Brunt's system or a comparable staging system.
- The studies reported the essential data, and the true positive (TP), false positive (FP), false negative (FN), and true negative (TN) were extracted so that a 2×2 table could be created. The authors of the primary research were contacted for additional information, if necessary.
- Different cohorts from a primary study not containing overlapping data were analyzed.
- Data not written in English were excluded.
- Conference abstracts were excluded because quality assessment could not be performed.
- Both prospective and retrospective studies were acceptable. Studies that reported other non-invasive methods were also allowed if the discrete data for RET could be extracted.

Two reviewers (HS.H. and J.L.) assessed the journals independently by using the criteria as shown above. When discrepancies surfaced, a final consensus opinion was adopted after discussion or in consultation with a third investigator (Q.L.).

### Data Synthesis and Statistical Analysis

From the 2×2 tables, summary sensitivity, specificity and diagnosis odds ratio (DOR) (with corresponding 95% confidence interval) were calculated. The DOR expresses how much greater the odds are of the patients being diagnosed correctly rather than the patients with misdiagnosis [27]. Meanwhile, AUROC derived from the data shows the overall effectiveness of each quantitative method. The heterogeneity in each stage of hepatic fibrosis of DOR was evaluated by performing Higgins's I^2^ and χ^2^ tests. The random- effects model was used for meta-analysis if there was significant heterogeneity existing among studies. Otherwise, the fixed effect model was applied. The random-effects model incorporated heterogeneity of studies in the analysis of the overall efficacy of RTE in the different studies [28]. And Deeks' funnel plot asymmetry test was performed to evaluate the publication bias.

To examine the potential sources of heterogeneity, the following covariates were predefined: etiology (viral hepatitis vs. not), ultrasound experiment (EU7500 vs. EUB8500 vs. HIVISION900 vs. HIVISION Preirus), and study quality factors (yes vs. unclear vs. no, for the individual QUADAS item as described below).

Pre-test probabilities of 25%, 50%, and 75% were assumed. The corresponding post-test probabilities were calculated following a “positive” or “negative” RTE result based on the summary sensitivity and specificity, which showed the relationship among the prior probability specified, the likelihood ratio, and posterior test probability [29]. “Positive” results were defined as all results above the optimal liver stiffness threshold given in each of the studies, while “negative” test results were defined as all results below the same threshold [30]. Statistical analyses were performed using STATA 12.0, particularly the meta-disc 14.0. All statistical tests were two-sided, with a *p* value <0.05 indicating statistical significance.

### Quality Assessment

The methodological quality of each study was assessed using a checklist based on the Quality Assessment for Studies of Diagnostic Accuracy (QUADAS) questionnaire [31]. There are fourteen items in the QUADAS questionnaire which were rated as yes, no, or unclear. Two investigators (HS.H. and J.L.) performed a quality assessment of the included studies independently, and discrepancies were resolved by discussion or in consultation with a third investigator (Q.L.).

## Results

### Selection of Candidate Studies

**Figure 1:**
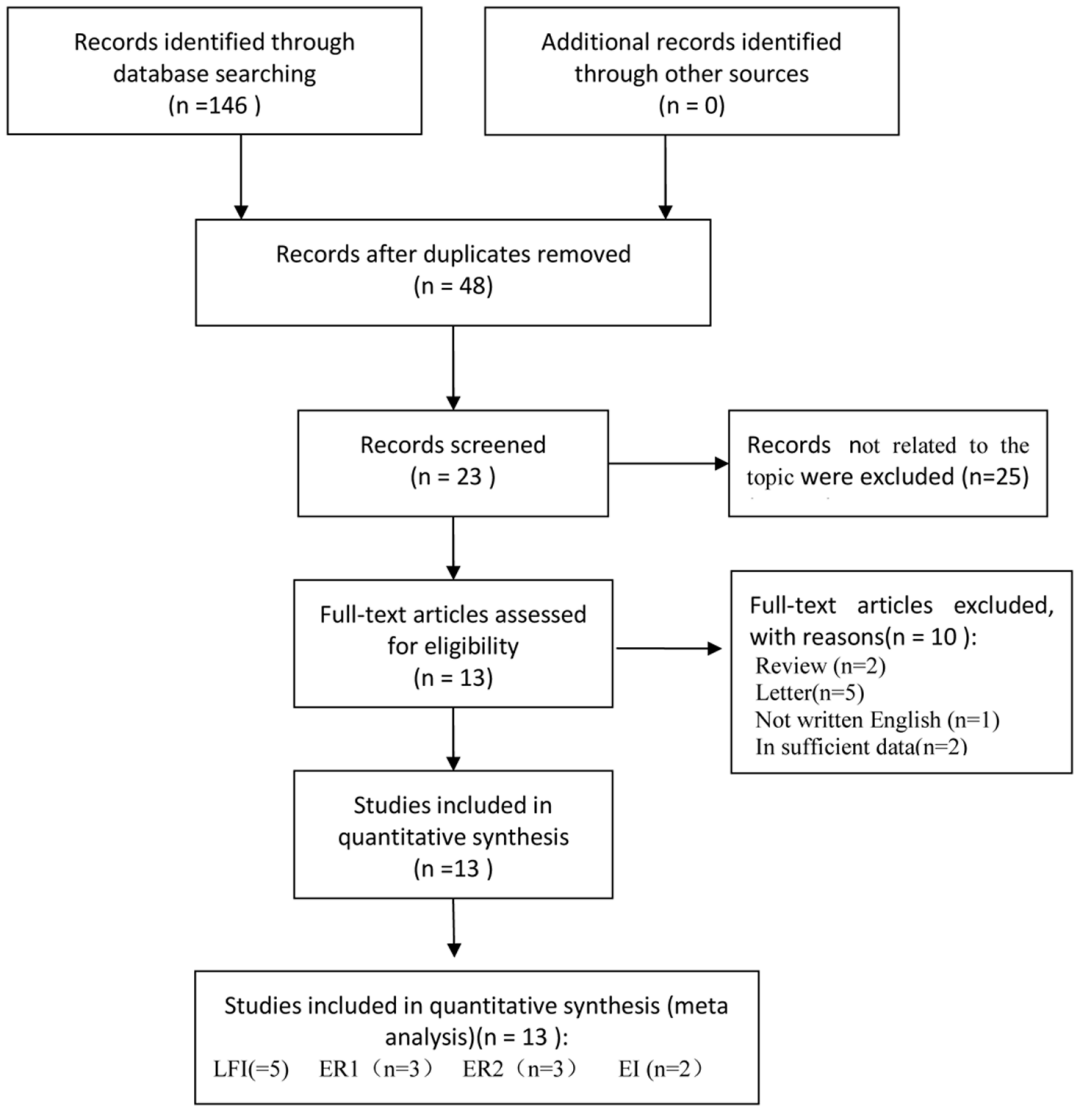
Flow diagram of search results and study selection.

48 citations were initially after the removal of duplicates, with 13 studies ultimately identified as meeting all inclusion criteria. A study by Ochi et al. [15] was used as two studies because the study divided the subjects into a training set and a validating set between which there were no overlapping data. Thirty-five studies were excluded for undesirable article types (n = 25), not written in English (n = 1), Review (n = 2), Letter (n = 5) and insufficient data (n = 2) (Fig. 1). All 13 studies fulfilled >10/14 QUADAS items and successfully passed the quality assessment (S1 Table).

### Patient Characteristics and Study Results

The 13 evaluated studies involved 1,347 patients with a mean age of 51.5 years. A final number of five articles for LFI [22], [32]–[35], three articles for ER1 [15], [36], [37], three articles for ER2 [20], [24], [38], two articles for EI [19], [23] were assessed to be suitable for inclusion in the meta-analysis. However, statistical analysis was not possible on the EI data due to there being too few studies. The fibrosis staging systems used to classify liver histology were varied. Ten studies (76.9%) used the METAVIR score, two (15.4%) studies used the Brunt's system and one (7.7%) study used the Scheuer score. The main characteristics of the included studies are shown in Tables 1 and 2.

**Table 1:** Characteristics of studies evaluating the performance of real time elastography for staging liver fibrosis.

Author year	Ref.	Country	Patients	Male(%)	Disease etiology	RTE measurement
Fujimoto 2013	22	Japan	310	46.8	CHC	LFI
Chung 2013	28	Korea	74	47.3	CHB,CHC,ALD, NAFLD,AIH,Toxic hepatitis	LFI
Tamaki 2013	29	Japan	115	59.1	CHC	LFI
Ferraioli 2012	30	Italy	130	70.0	CHC	LFI
Tomeno 2013	31	Japan	93	44.1	CHC	LFI
Hu 2014	32	China	75	66.7	CHB	ER1
Koizumi 2011	33	Japan	70	65.7	CHC	ER1
Ochi 2012	15	Japan	106	50.9	NAFD	ER1
Ochi 2012	15	Japan	75	54.7	NAFD	ER1
Paparo 2013	20	Italy	60	56.7	Disease with liver iron overload	ER2
Kanamoto 2009	34	Japan	41	73.2	CHB CHC	ER2
Xie 2012	24	China	71	60.6	CHB	ER2
Wang 2010	23	China	55	58.2	CHB	EI
Colombo 2012	19	Japan	72	61.1	CHB,CHC,ALD, NAFLD,AIH,PBC	EI

RTE, real time elastography; LFI, liver fibrosis index; ER1, the elastic ratio of the liver for the intrahepatic venous; ER2, the elastic ratio of the liver for the intercostal muscle; EI, elastic ratio; CHB, chronic hepatitis B; CHC, chronic hepatitis C; ALD, alcoholic liver disease, NAFLD, nonalcoholic liver fatty disease; AIH, autoimmune hepatitis; PBC, primary biliary cirrhosis.

**Table 2:** Diagnostic indices of studies evaluating the performance of RTE for staging liver fibrosis.

		Fibrosis F≥2		Fibrosis F≥3		Fibrosis F = 4	
Study	RTE measurement	Cut-off	Sensitivity/Specificity (%)	Cut-off	Sensitivity/Specificity (%)	Cut-off	Sensitivity/Specificity (%)
Fujimoto	LFI	1.92	78.6/78.0	NA	NA/NA	2.56	79.2/80.5
Chung	LFI	2.54	64.9/35.3	NA	NA/NA	2.79	81.0/64.2
Tamaki	LFI	N/A	70.3/84.3	NA	90.6/71.1	NA	NA
Ferrailoli	LFI	1.82	81.7/60.0	1.86	91.7/57.4	2.33	66.7/84.0
Tomeno	LFI	2.39	90.2/44.2	2.62	92.3/46.2	3.59	100/78
Hu	ER1	2.62	86.2/88.2	3.20	91.4/85.0	3.86	94.1/82.8
Koizumi	ER1	2.73	82.8/91.7	3.25	85.7/96.4	3.93	91.3/91.5
Ochi	ER1	2.67	86.0/88.7	3.02	88.2/91.5	3.36	100/85.6
Ochi	ER1	2.67	92.3/89.8	3.02	88.9/96.5	3.36	100.0/95.3
Paparo	ER2	NA	NA/NA	2.75	70.0/97.5	2.75	87.5/84.6
Kanamoto	ER2	1.18	96.2/73.3	0.75	95.5/89.5	0.60	93.3/73.1
Xie	ER2	1.10	77.8/80.0	0.75	61.5/91.1	0.60	50.0/96.7
Wang	EI	55.33	81.6/88.2	80.71	73.1/75.0	90.31	71.4/80.0
Colombo	EI	1.89	76.0/66.0	NA	NA/NA	3.60	80.0/90.3

RTE, real time elastography; LFI, liver fibrosis index; ER1, the elastic ratio of the liver for the intrahepatic venous; ER2, the elastic ratio of the liver for the intercostal muscle; EI, elastic index; NA, not available.

### Meta-Analysis of RTE for Staging Liver Fibrosis

**Figure 2:**
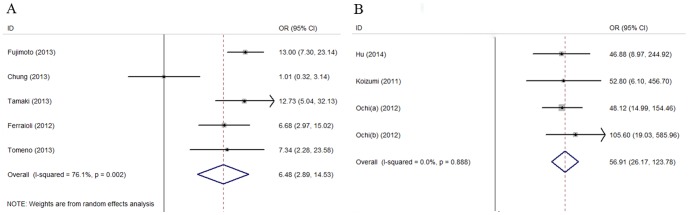
Forest plot from meta-analysis of DOR value using a random-effect or fixed-effect model for significant fibrosis. (A) Forest plot of LFI and (B) Forest plot of ER1. DOR: diagnostic odds ratio; LFI: liver fibrosis index; ER1: the elastic ratio of the liver for the intrahepatic vein; Ochi (a): the training set of the subjects in the study by Ochi et al; Ochi (b): the validating set of the subjects in the study by Ochi et al.

For predicting significant fibrosis (F≥2), the summary sensitivities of LFI and ER1 were 0.78 (95% CI, 0.70–0.84) and 0.86 (95% CI, 0.80–0.90), respectively. The specificities were 0.63 (95% CI, 0.46–0.78) and 0.89 (95% CI, 0.83–0.94), respectively. The summary DOR were 6.48 (95% CI, 2.89–14.53) and 56.91 (95% CI, 26.17–123.78), respectively (Fig. 2). The AUROC were 0.79(95% CI, 0.75–0.82)for LFI and 0.94(95% CI, 0.92–0.96)for ER1. There was statistically significant heterogeneity for LFI of DOR (*p* = 0.002, *I* ^2^ = 76.1%). According to the meta-regression analysis, the main source of heterogeneity was etiology (*p* = 0.032). However, There was no statistically significant heterogeneity for ER1 of DOR (*p* = 0.888, *I* ^2^ = 0.64%). In the stage of significant fibrosis, the number of studies on ER2 is too small to be included for meta-analysis.

**Figure 3:**
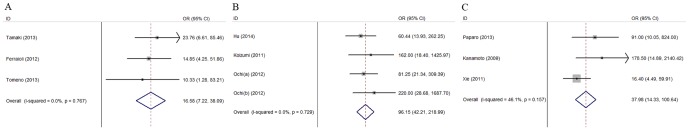
Forest plot from meta-analysis of DOR value using a random-effect or fixed-effect model for significant fibrosis. (A) Forest plot of LFI and (B) Forest plot of ER1 and (C) Forest plot of ER2. DOR: diagnostic odds ratio; LFI: liver fibrosis index; ER1: the elastic ratio of the liver for the intrahepatic vein; ER2: the elastic ratio of the liver for the intercostal muscle; Ochi (a): the training set of the subjects in the study by Ochi et al; Ochi (b): the validating set of the subjects in the study by Ochi et al.

For predicting advanced fibrosis (F≥3), the summary sensitivity of LFI, ER1, and ER2 were 0.91 (95% CI, 0.83–0.96), 0.88 (95% CI, 0.81–0.93) and 0.75 (95% CI, 0.63–0.85), respectively. The specificity were 0.58 (95% CI, 0.52–0.64), 0.93 (95% CI, 0.87–0.96) and 0.93 (95% CI, 0.87–0.97), respectively. The summary DOR were 16.58 (95% CI, 7.22–38.09), 96.15 (95% CI, 41.21–218.99) and 37.98 (95% CI, 14.33–100.64), respectively (Fig. 3). There were no statistically significant heterogeneities of these three pooled DOR (*p* = 0.7667, *I* ^2^ = 0.0%), (*p* = 0.729, *I* ^2^ = 1.30%) and (*p* = 0.158, *I* ^2^ = 3.69%), respectively. The AUROC were 0.95 for LFI, 0.93 for ER1 and 0.96 for ER2.

**Figure 4:**
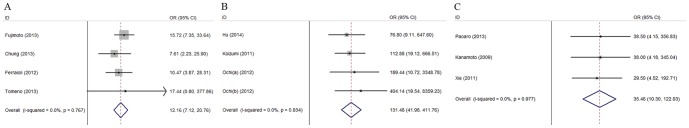
Forest plot from meta-analysis of DOR value using a random-effect or fixed-effect model for significant fibrosis. (A) Forest plot of LFI and (B) Forest plot of ER1 and (C) Forest plot of ER2. DOR: diagnostic odds ratio; LFI: liver fibrosis index; ER1: the elastic ratio of the liver for the intrahepatic vein; ER2: the elastic ratio of the liver for the intercostal muscle; Ochi (a): the training set of the subjects in the study by Ochi et al; Ochi (b): the validating set of the subjects in the study by Ochi et al.

For predicting cirrhosis (F = 4), the summary sensitivity of LFI, ER1, and ER2 were 0.79 (95% CI, 0.61–0.91), 0.96 (95% CI, 0.87–0.99) and 0.79 (95% CI, 0.61–0.91), respectively. The specificity were 0.88 (95% CI, 0.81–0.93), 0.89 (95% CI, 0.83–0.93) and 0.88 (95% CI, 0.81–0.93), respectively. The summary DOR were 12.16 (95% CI, 7.12–20.76), 131.48 (95% CI, 41.98–411.76) and 35.46 (95% CI, 10.30–122.03), respectively (Fig. 4). There were no statistically significant heterogeneities of pooled OR (*p* = 0.767, *I* ^2^ = 0.0%) (*p* = 0.15, *I* ^2^ = 44.27%) (*p* = 0.977, *I* ^2^ = 0.0%). The AUROC were 0.85 (95% CI, 0.81–0.87) for LFI, 0.93 (95% CI, 0.94–0.98) for ER1, and 0.92 for ER2.

Summary estimations for EI could not be performed in this study due to insufficient data being available, with only two studies that fit our criteria. The AUROC for advanced fibrosis (F≥3) were 0.75 and 0.92, respectively and for cirrhosis (F = 4) were 0.66 and 0.85, respectively, across a heterogeneous range of liver disease.

### Publication Bias

According to Deeks'funnel plot asymmetry test, there was no publication bias among the studies of LFI, ER1, ER2 for each stage of hepatic fibrosis (*P*>0.05).

### Fagan Plot Analysis

The Fagan plot in LFI demonstrated that the negative post-probabilities of significant fibrosis (F≥2) were 11%, 26%, 52%, respectively, and the positive post-probability ranged from 46% to 86% when the pre-test probability was 25% or 50% or 75% (S1 Figure). For F = 4, the negative post-probabilities were 9%, 22%, 46%, respectively, and the positive post-probability ranged from 54% to 91% when the pre-test probability was 25% or 50% or 75% (S2 Figure).

With regard to ER1, the results showed that the negative post-probabilities of significant fibrosis (F≥2) were 5%, 14%, and 32%, respectively, and the possibility of a precise diagnosis for patients with positive results ranged from 73% to 96% when the pre-test probability was 25%, 50%, 75% (S3 Figure). For F≥3, the negative post-probabilities were 4%, 11%, 27%, respectively, and the positive post-probability ranged from 80% to 92% when the pre-test probability was 25%, 50%, 75% (S4 Figure). As to cirrhosis (F = 4), the negative post-probabilities were 2%, 5%, and 13%, respectively, and the possibility of an accurate diagnosis with a “positive” measurement ranged from 74% to 96% when the pre-test probability was 25%, 50%, 75% (S5 Figure).

Due to insufficient data, the Fagan plot was not performed for ER2 and EI.

## Discussion

To overcome the limitations of LB, great efforts have been made to develop and validate noninvasive methods for detecting liver fibrosis, including serological indicators and imaging methods. Among these noninvasive methods, ultrasound technology has been developed rapidly over the years. Especially, transient elastography (TE) based on ultrasonic elastography principles, has been validated in many previous studies; some meta-analysis on TE showed that the method had good performance in the assessment of hepatic fibrosis [30], [39]–[41]. However, Arena et al. reported that necro-inflammatory activity strongly and independently influenced TE measurement in patients who did not have cirrhosis [42]. In addition, the reproducibility of TE was reportedly lower in patients with steatosis, increased body mass index (BMI), lower degrees of hepatic fibrosis and severe ascites [43]. Different from TE, RTE evaluates the degree of liver fibrosis through a slight compression of body tissue that induces a strain into the tissues. Koizumi et al. reported that skin fold thickness, BMI, and liver steatosis were not identified as factors affecting the elastic ratio determined. [37] On the other hand, RTE measures the deformation of the tissue examined in conventional B-mode ultrasound imaging, thus RTE enables the display of anatomical structures while measuring the stiffness of the liver. With the development of RTE, different quantitative methods according to the technology were explored as described above. Our study evaluated the overall effectiveness of these methods to guide clinical practice. As far as we know, this study is the first to explore the pooled performance of RTE.

The overall results suggested that LFI was excellent in diagnosing F≥3 (AUROC 94.53%, sensitivity 91%, specificity 68%) and has moderate accuracy for F≥2 (AUROC 79%, sensitivity 78%, specificity 63%), F = 4 (AUROC 85%, sensitivity 77%, specificity 78%). Meanwhile, Fagan plot analysis was performed for F≥2 and F = 4 to evaluate the clinical utilities of LFI. However, our results showed that LFI could not be applied to accurately differentiate F≥2 versus F0-1 and F = 4 versus F0-3. In stage of F≥2, when the pre-test probability = 50%, there was only 68% probability of correctly diagnosing F≥2 with a “positive” result; however, the diagnosis would be wrong in 26% of the patients with a negative measurement. For F = 4, when the pre-test probability  = 50%, 78% of the patients following “positive” results were correctly diagnosed, while the diagnosis would be wrong in 22% of the patients with a “negative” measurement. With regard to F≥3, Fagan plot analysis was not performed due to insufficient data (only three studies were included). In addition, significant heterogeneity was present in the assessment of significant fibrosis (F≥2, *p* = 0.032). Etiology was found as the main source for heterogeneity through meta-regression analysis; thus, the pooled performance was calculated with a random-effects model. According to the results of heterogeneity, further studies of RTE for the assessment of viral hepatitis versus non-viral liver disease are required in the future. Compared with the pooled performance of TE [30], [39], LFI seems to have no potential to substitute for TE in the assessment of liver fibrosis.

Another equation studied by Juan Wang et al. and Colombo et al. denoted as EI, showed that the AUROC ranged from 0.75 to 0.92 for the diagnosis of F≥2 and 0.66 to 0.85 for the diagnosis of F = 4. The utility of this quantitative method could not be concluded since there are too few studies. Thus, additional studies are required to explore the performance of EI.

Compared to LFI and EI that are generated through quantitative equations based on elastic parameters, ER1 and ER2 were also explored and validated in studies previously. As previously stated, the AUROC values of ER1 for diagnosing F≥2, F≥3 and F = 4 were 0.94, 0.95, and 0.96, respectively, when the intrahepatic small vessel was chosen as the reference. The data diagnosing liver fibrosis was excellent in diagnosing test. Fagan plot analysis was used to explore the utility of ER1. In the group of F≥2, when the pre-test probability was 50%, ER1 accurately diagnosed liver fibrosis in 89% of the patients with a “positive” measurement and misdiagnosis was present in only 14% of patients following a negative result. For F≥3, when the pre-test probability was 50%, there was more than a 90% probability in correctly diagnosing hepatic fibrosis following a “positive”measurement and the diagnosis would be incorrect in only 11% of the patients with a “negative ” measurement. For F = 4, when the pre-test probability was 50%, there was a 90% probability of correctly diagnosing hepatic fibrosis with a positive result, and the diagnosis would be wrong in only 5% of the patients with a negative value. The combined diagnostic effects for each stage of hepatic fibrosis were excellent and comparable or even superior to the results reported in previous meta-analysis of TE. No significant heterogeneity was found in any degree of fibrosis on ER1. However, the study by Ochi et al., which was included in our meta-analysis, divided the subjects into a training set and a validation set [15]. These two groups of patients were analyzed separately in our statistical analysis, which might affect the results of heterogeneity. According to the excellent performance of ER1, we recommend that the elastic ratio of the liver for the intrahepatic vein (ER1) be applied to clinical practice.

Though both of the results for ER1 and ER2 were shown as the elastic ratio, Kanamoto et al. chose the intercostal muscle as the reference and the elastic ratio was defined as the value of the liver parenchyma divided by the reference value [38]. Due to insufficient data, the pooled performance in F≥2 could not be calculated. The overall results suggest that ER2 was good in diagnosing F≥3 (AUROC 95.89%, sensitivity 75%, specificity 93%) and F = 4 (AUROC 92.2%, sensitivity 79%, specificity 88%). Though the combined effects of ER2 were acceptable, we would not conclude that the methods would be equal to TE. Only three studies and 172 patients were included in statistical analysis. Paparo et al. defined the elastic ratio as the intercostal muscle value divided by the value of the liver parenchyma opposed to Kanamoto and Xie. Meanwhile, the RTE module used by Paparo et al. differed from that produced by Hitachi due to a shortest range of pixel's values (i.e. from 0–100 versus the Hitachi 256 step wise grading) to represent strain distribution and tissue elasticity. Moreover, the liver fibrosis was diagnosed by TE and not liver biopsy in the study by Paparo et al. However, there was no significant heterogeneity according to our statistical analysis. The main reason may be that the principle of the elastic ratio is the same despite which high-end ultrasound system from different manufactures was used. Above all, we draw the conclusion that the elastic ratio of the liver for the intercostal (ER2) is a promising method for the diagnosis of liver fibrosis, although further studies with a large sample size are required.

In spite of the good or excellent performance for staging liver fibrosis, RTE also has an obvious shortcoming. As the principle of RTE is measuring the deformation created by the compression, different magnitudes of the pressure applied may cause discrepant displacement even in the same patient. Compared with Siemens, Philips, and Toshiba, the ultrasound machine used to perform RTE with the Hitachi system has a wider range of applications. Both the Hitachi EUB 8500 and EUB900 performed RTE with manual compression inducing intra- and inter-observer variability. To overcome the shortcoming, a new quantitative analysis called the Hitachi Hi Vision Preirus was applied to the clinic. It can acquire strain image by tissue compression from the beat of heart. Consequently, the external pressure is standard. Then, the new ultrasonic system can reduce intra-and inter- observer variability.

Some limitations of this study should be taken into consideration. First, in our meta-analysis, results were generated from different etiological groups within the same analysis. Chronic hepatitis with different pathogens may affect the progression of the hepatic fibrosis in different ways and result in various images generated through RTE. We encourage investigators to be rigorous in their patient selection in future studies. In addition, a comparative study among the underlying liver diseases with RTE is required. Significant heterogeneity was not present in the evaluation of the different quantitative measurements except for in the group F≥2 for LFI. There were likely too few studies included in the meta-analysis.

In conclusion, our meta-analysis shows that the elastic ratio of the liver for the intrahepatic vein seems to have more excellent performance compared to TE as a non-invasive method in assessment of liver fibrosis. However, we just made the conclusion from the data which described in this article and some meta-analysis on TE. A head-to-head comparative study is urgently needed to prove this point. The elastic ratio of the liver for the intercostal muscle is also a promising method to be applied to diagnose liver fibrosis; however, additional studies are required to validate the conclusion. Meanwhile, according to the results of the Fagan plot analysis, LFI and EI are not good enough to apply to clinical practice. In the future, it is necessary to further evaluate the potential value of RTE in a large, prospective, international, multi-center study.

## Supporting Information

S1 Figure **Fagan plot analysis to evaluate the clinical utility of LFI for F≥2.**
(TIF)

S2 Figure **Fagan plot analysis to evaluate the clinical utility of LFI for F = 4.**
(TIF)

S3 Figure **Fagan plot analysis to evaluate the clinical utility of ER1 for F≥2.**
(TIF)

S4 Figure **Fagan plot analysis to evaluate the clinical utility of ER1 for F≥3.**
(TIF)

S5 Figure **Fagan plot analysis to evaluate the clinical utility of ER1 for F = 4.**
(TIF)

S1 Table **Quality assessment of included studies.**
(XLS)

S1 Checklist **PRISMA checklist of the meta-analysis.**
(DOC)
