# Supplementary figures and images for: Performance of Real-Time Elastography for the Staging of Hepatic Fibrosis: A Meta-Analysis
Source: PLoS One. 2014 Dec 26;9(12):e115702. doi: 10.1371/journal.pone.0115702 (PMC4277316; doi:10.1371/journal.pone.0115702)

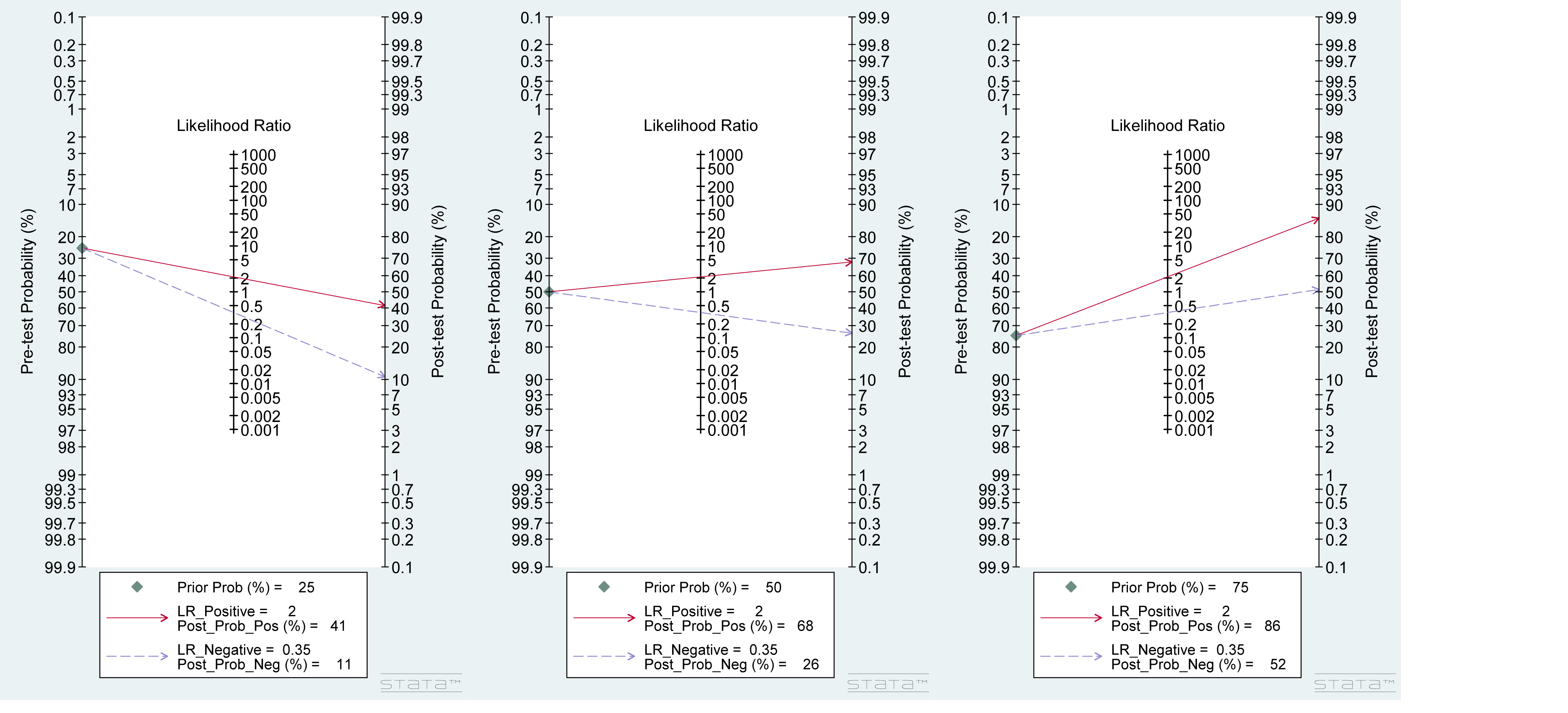

Supplement: S1 Figure — Fagan plot analysis to evaluate the clinical utility of LFI for F≥2. (TIF) [file pone.0115702.s001.tif]

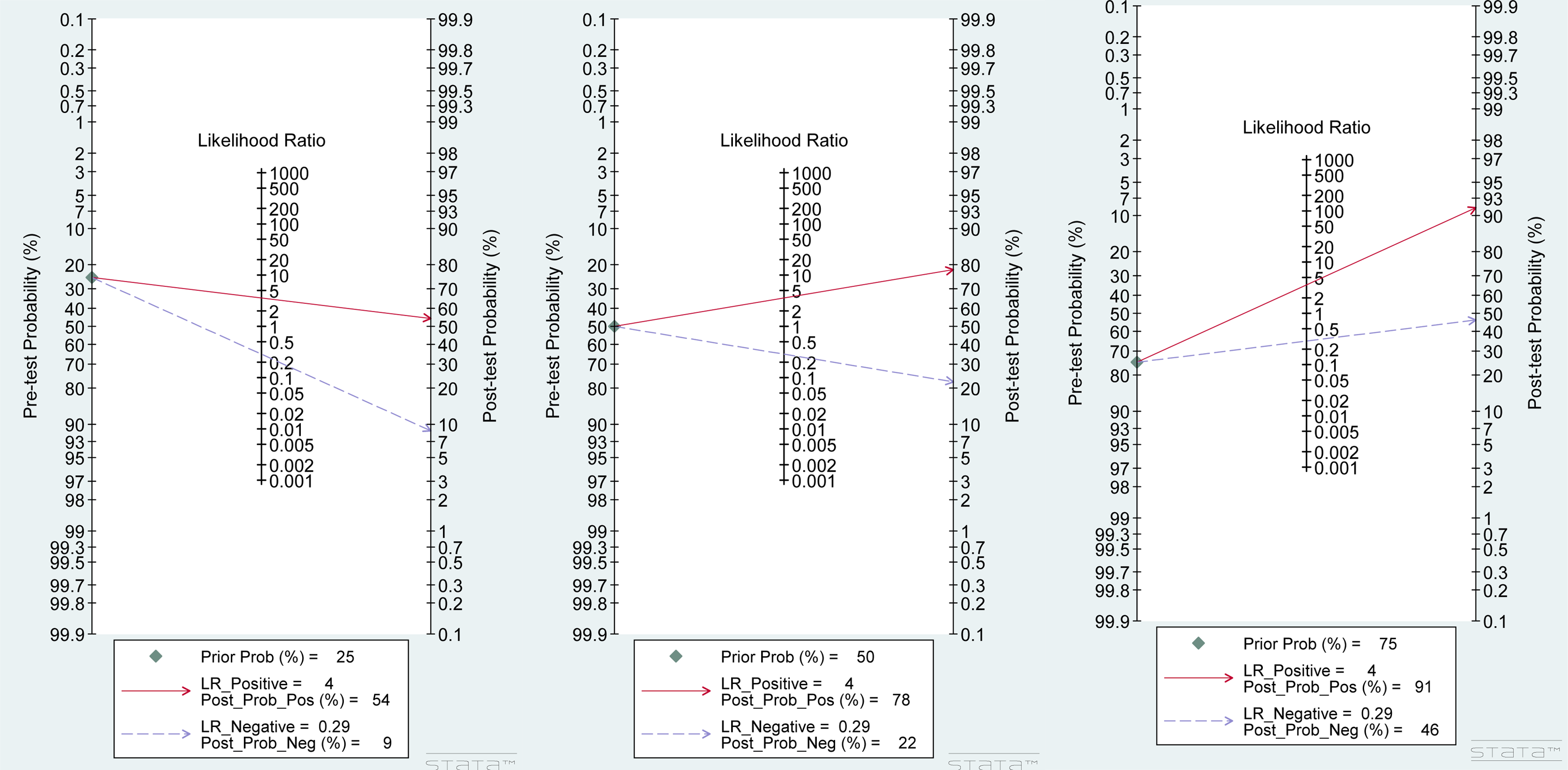

Supplement: S2 Figure — Fagan plot analysis to evaluate the clinical utility of LFI for F = 4. (TIF) [file pone.0115702.s002.tif]

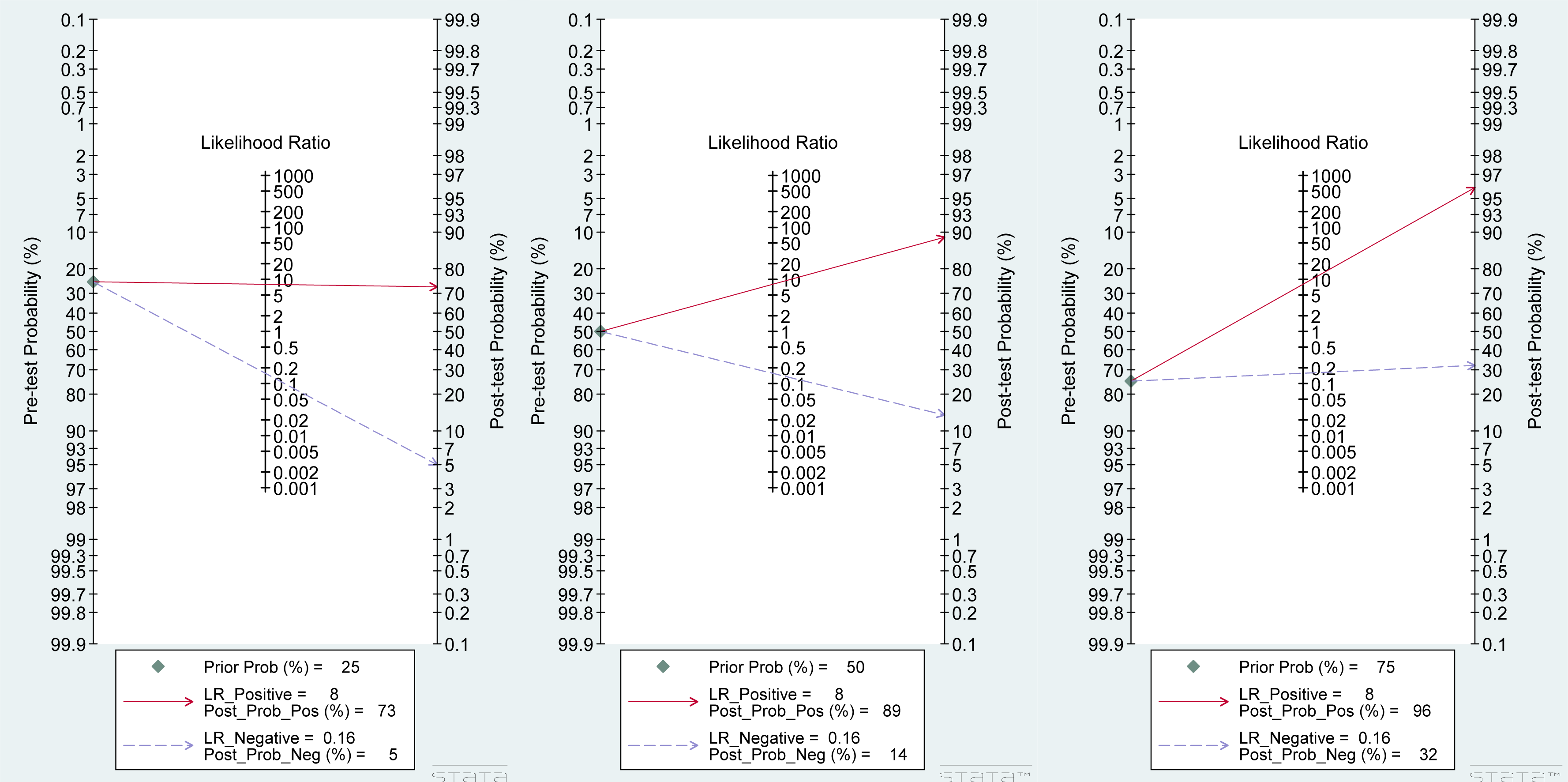

Supplement: S3 Figure — Fagan plot analysis to evaluate the clinical utility of ER1 for F≥2. (TIF) [file pone.0115702.s003.tif]

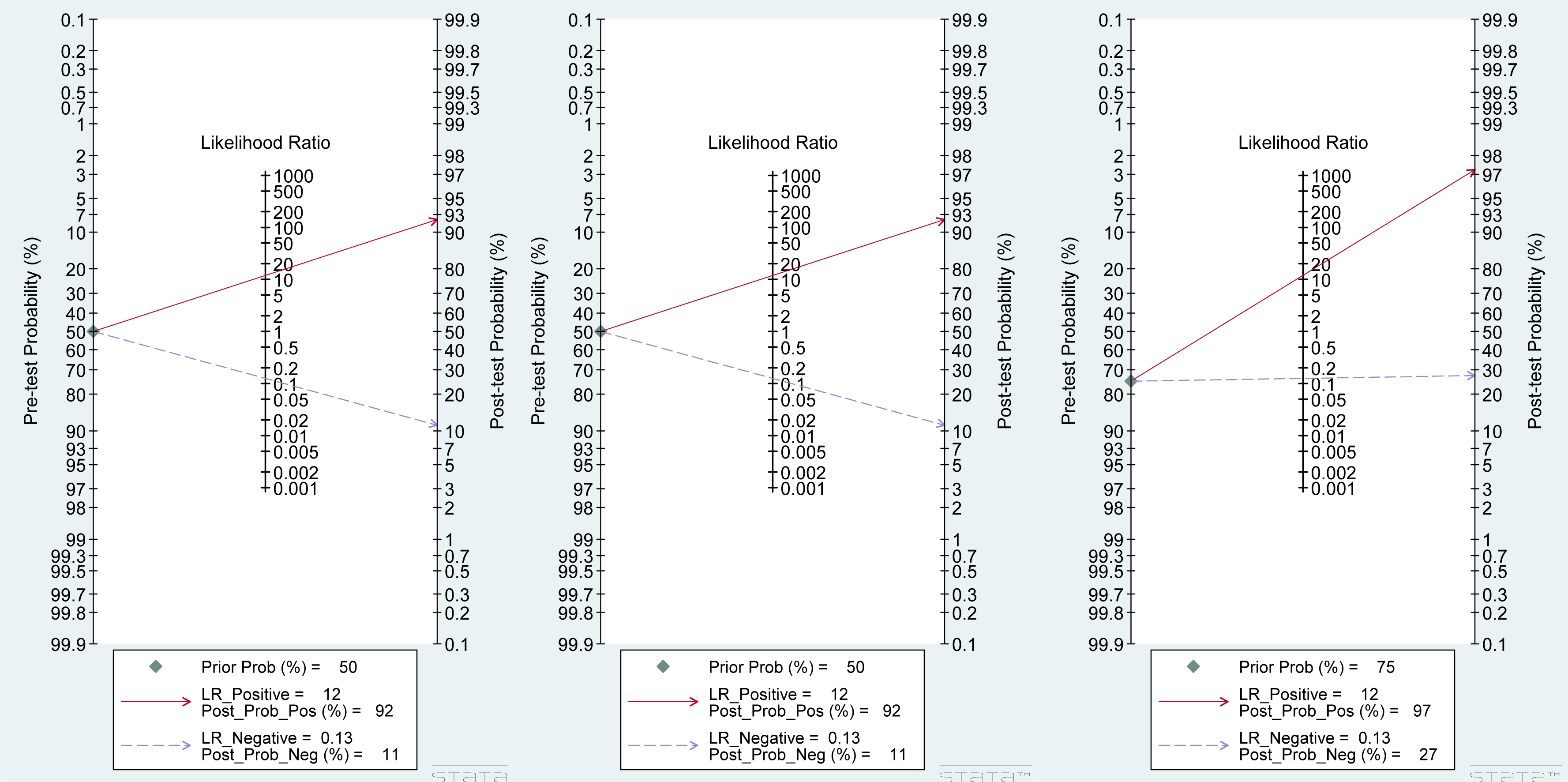

Supplement: S4 Figure — Fagan plot analysis to evaluate the clinical utility of ER1 for F≥3. (TIF) [file pone.0115702.s004.tif]

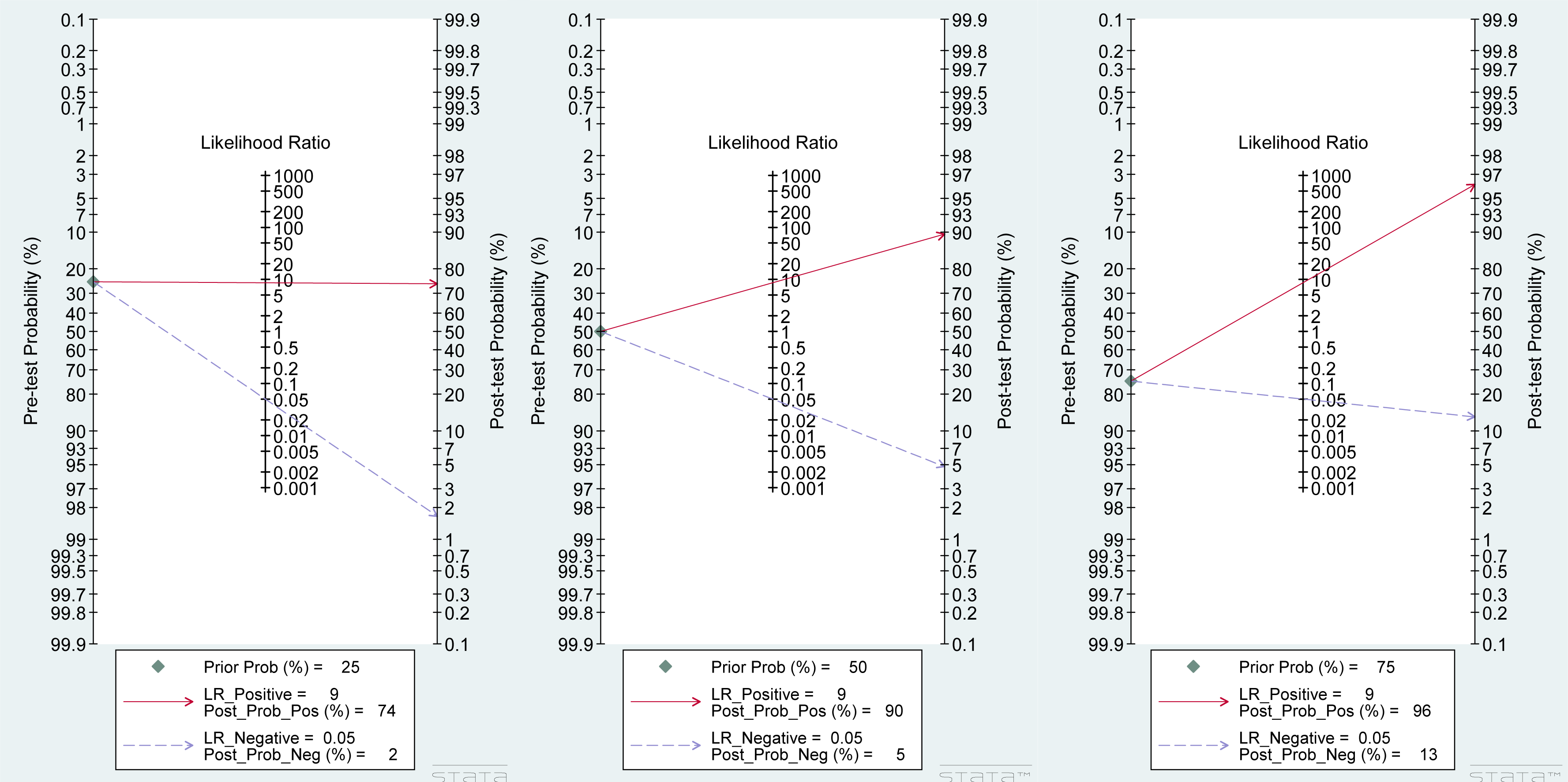

Supplement: S5 Figure — Fagan plot analysis to evaluate the clinical utility of ER1 for F = 4. (TIF) [file pone.0115702.s005.tif]
